# Genome-wide reconstitution of chromatin transactions reveals that RSC preferentially disrupts H2AZ-containing nucleosomes

**DOI:** 10.1101/gr.243139.118

**Published:** 2019-06

**Authors:** Aylin Cakiroglu, Cedric R. Clapier, Andreas H. Ehrensberger, Elodie Darbo, Bradley R. Cairns, Nicholas M. Luscombe, Jesper Q. Svejstrup

**Affiliations:** 1 Bioinformatics and Computational Biology Laboratory, The Francis Crick Institute, London NW1 1AT, United Kingdom;; 2 Department of Oncological Sciences, Huntsman Cancer Institute, and Howard Hughes Medical Institute, University of Utah School of Medicine, Salt Lake City, Utah 84112, USA;; 3 Mechanisms of Transcription Laboratory, The Francis Crick Institute, London NW1 1AT, United Kingdom;; 4 UCL Genetics Institute, Department of Genetics, Evolution and Environment, University College London, London WC1E 6BT, United Kingdom

## Abstract

Chromatin transactions are typically studied in vivo, or in vitro using artificial chromatin lacking the epigenetic complexity of the natural material. Attempting to bridge the gap between these approaches, we established a system for isolating the yeast genome as a library of mononucleosomes harboring the natural epigenetic signature, suitable for biochemical manipulation. Combined with deep sequencing, this library was used to investigate the stability of individual nucleosomes and, as proof of principle, the nucleosome preference of the chromatin remodeling complex, RSC. This approach uncovered a distinct preference of RSC for nucleosomes derived from regions with a high density of histone variant H2AZ, and this preference is indeed markedly diminished using nucleosomes from cells lacking H2AZ. The preference for H2AZ remodeling/nucleosome ejection can also be reconstituted with recombinant nucleosome arrays. Together, our data indicate that, despite being separated from their genomic context, individual nucleosomes can retain their original identity as promoter- or transcription start site (TSS)-nucleosomes. Besides shedding new light on substrate preference of the chromatin remodeler RSC, the simple experimental system outlined here should be generally applicable to the study of chromatin transactions.

The eukaryotic genome is stored as a polymer of nucleosomes in which ∼147 base pairs (bp) of DNA are wrapped around a core of eight histone proteins separated by linkers of variable length (Kornberg 1974; Kornberg and Lorch 1999). Because these complexes encompass the entire genome, they serve as the substrate and platform for all nuclear processes involving DNA. Nucleosomes influence reactions on DNA in at least two ways: By occluding access to the DNA, they can exert an inhibitory function; and by recruiting enzymes and regulatory factors, they may stimulate catalysis. The regulatory capacity of nucleosomes is amplified vastly through regional incorporation of an extensive assortment of posttranslational histone marks and histone variants, in turn rendering every nucleosome unique (Strahl and Allis 2000; Turner 2000).

A long-standing challenge in the field of chromatin research has been the lack of a reconstituted genome-wide system for studying the role of epigenetic modifications in the context of their natural sequences. Current approaches typically involve either assembling chromatin in vitro from naked DNA and free histones, or studying it inside the cell, in vivo. Even though both approaches have substantially advanced our understanding of chromatin function in general, they also suffer some limitations: Reconstituted chromatin has virtually none of the complexity of natural chromatin, being devoid of the natural genomic sequences and/or the combinatorial assortment of histone marks. Conversely, chromatin studied in vivo obviously retains its natural composition but is trapped in a context in which a process of interest is continuously subjected to the direct or indirect influence of the numerous biochemical reactions taking place inside the cell. Protocols in which chromatin is isolated directly from the host organism in a manner suitable for biochemical reconstitution have been described, but they are generally limited to the study of one or a few loci at a time (Griesenbeck et al. 2003; Unnikrishnan et al. 2012; Hamperl et al. 2014; Ehrensberger et al. 2015). As such, a system in which an entire genome is available in native form for the biochemical reconstitution of chromatin transactions would be very useful.

RSC (Remodels the Structure of Chromatin) is a member of the Swi2/Snf2 family of ATP-dependent chromatin remodelers and the most abundant chromatin remodeling factor in yeast (Cairns et al. 1996). It is necessary for transcription by all three nuclear RNA polymerases (Parnell et al. 2008) and contributes to the establishment of the canonical nucleosome-depleted or ‘nucleosome-free’ region (NFR) found in the majority of yeast RNAPII promoters (Hartley and Madhani 2009; Wippo et al. 2011; Lorch et al. 2014). By itself, RSC destabilizes nucleosomes such that their DNA becomes sensitive to nuclease digestion (Cairns et al. 1996). In the presence of a histone acceptor such as naked DNA or the histone chaperone Nap1, RSC can fully disassemble mononucleosomes into naked DNA (Lorch et al. 1999, 2006), but it can also eject nucleosomes from nucleosome arrays even in the absence of NAP1 (Clapier et al. 2016). The mechanism of RSC action has been studied through a variety of biochemical and structural approaches and involves RSC conducting directional DNA translocation from a site within the nucleosome, pumping DNA around the octamer, resulting in nucleosome sliding and/or ejection of either the RSC-bound octamer or the one adjacent to it (Saha et al. 2005; Chaban et al. 2008). Despite the abundance of data on its mechanism, the mode by which RSC is targeted to specific loci remains unclear. It can be recruited by transcription factors (Swanson et al. 2003; Inai et al. 2007), but it also harbors its own DNA-binding domains (Angus-Hill et al. 2001; Badis et al. 2008) and eight bromodomains, at least one of which has been implicated in recruitment to acetylated histones (Kasten et al. 2004). RSC has been shown to selectively remodel at the promoter but not the open-reading frame nucleosomes on purified *PHO5* gene rings (Lorch et al. 2011). It also shows a preference for nucleosomes bearing poly(dAdT) tracts in vitro (Lorch et al. 2014). We chose to work with RSC as the model enzyme to validate the functionality of genomic nucleosomes for several reasons: (1) When combined with NAP1, the reaction product, naked DNA, can easily be separated from mononucleosomes that are not remodeled for deep sequencing in order to characterize the underlying DNA; (2) the enzyme is abundant and the reaction robust; and (3) RSC plays important roles in transcription (Parnell et al. 2008, 2015). The purpose of our approach was to identify nucleosomes that were disassembled preferentially above the generic background of RSC activity in the hope that we would gain new insights into the catalytic preferences of this important chromatin remodeler.

Histone H2AZ (encoded by the nonessential *HTZ1* gene in budding yeast) is a variant of histone H2A that shares 60% sequence identity with its canonical H2A counterpart (Suto et al. 2000). While H2AZ localizes almost exclusively to promoters (Zhang et al. 2005) and often occupies the two nucleosomes surrounding the NFR (Raisner et al. 2005), yeast cells lacking H2AZ still contain intact NFRs (Hartley and Madhani 2009). Even though H2AZ localizes to virtually all promoters, irrespective of expression level (Raisner et al. 2005), its occupancy differs between promoters (Albert et al. 2007). When genes are dynamically activated, H2AZ occupancy decreases, suggesting a poising function that might assist in gene activation (Zhang et al. 2005). Incorporation of H2AZ is catalyzed by the chromatin remodeler SWR1 by replacement of the H2A/H2B dimer on a canonical nucleosome with a H2AZ/H2B dimer (Mizuguchi et al. 2004). Despite the distinctive localization of H2AZ and the comprehensive understanding of its deposition mechanism, relatively little is known about the function of H2AZ on the promoters on which it resides.

Nucleosome occupancies are determined not only by chromatin remodeling enzymes but also by the intrinsic physical stability of nucleosomes. Features such as high AT-content and histone acetylation have thus been correlated with decreased nucleosome stability in vitro (Li et al. 1993; Brower-Toland et al. 2005; Tillo and Hughes 2009) and decreased occupancy in vivo (Yuan et al. 2005; Kaplan et al. 2009).

Here, we show that a library of purified, native mononucleosomes isolated from yeast can be used to study the specificity of chromatin-modifying or -remodeling enzymes such as RSC.

## Results

### Purification of genomic chromatin and description of assays

**Figure 1.**
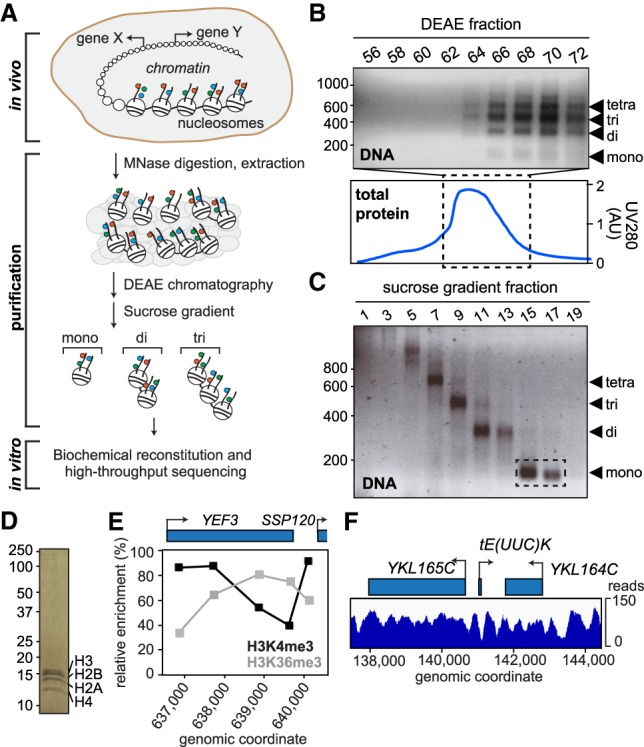
Purification and characterization of genomic chromatin. (*A*) Schematic of experimental approach. Colored circles, histone marks. (*B*) DNA from DEAE fractions analyzed by agarose gel electrophoresis (*top* panel); marker on left shows length in bp. *Bottom* panel, chromatogram of total eluted protein. Fractions 66–72 were loaded on the sucrose gradient. (*C*) DNA from sucrose gradient analyzed by agarose gel electrophoresis. Fractions used for experiments are indicated by stippled box. (*D*) Silver-staining of purified mononucleosomes from *C*. (*E*) Histone mark patterns of purified mononucleosomes as determined by native ChIP-qPCR. Histone marks were normalized to histone H3, as in the reference data sets of Pokholok et al. (2005) and Kim and Buratowski (2009). (*F*) Representative map of nucleosomes on Chromosome XI after paired-end sequencing and alignment to the yeast genome.

In order to generate a library of native yeast nucleosomes, we developed a three-step purification protocol (Fig. 1A): First, purified yeast nuclei were incubated with micrococcal nuclease (MNase), which preferentially digests naked DNA to generate short chromatin fragments. The resulting fragments were extracted from the nuclei, then bound to and eluted from DEAE sepharose (Fig. 1B). This was followed by ultracentrifugation through a sucrose gradient to separate the fragments by length to further remove contaminating proteins and free DNA. By adjusting the amount of MNase and the conditions of ultracentrifugation, it was possible to fine-tune the proportions of nucleosomal species, ranging in length from mono- to tetranucleosomes (Fig. 1C). Under such limiting conditions, little or no overdigestion of nucleosomes occurred. Indeed, these mononucleosomes were similar in nature to nucleosomes previously defined as ‘underdigested’ by Weiner et al. (2010). The final material used consists entirely of mononucleosomes and is of high purity, as judged by SDS-PAGE analysis (Fig. 1C,D). In vitro ChIP of selected histone marks across a previously characterized gene (Kim and Buratowski 2009) showed the expected profile (Fig. 1E), indicating that the epigenetic marks of the original material are indeed maintained. Paired-end sequencing of purified mononucleosomes followed by mapping of the reads to the yeast genome showed a typical nucleosomal pattern for the nucleosomes that RSC was presented with (Fig. 1F; see also Supplemental Fig. S1A). The majority of the genome was recovered in the purification: 94% was thus covered by at least five reads. As might be expected, areas with less, or no, reads were mainly found toward the gene-poor ends of the chromosomes (Supplemental Fig. S1B). This agrees with the previous finding that yeast chromatin is mostly open and active (Rattner et al. 1982) and shows that native nucleosomes are generally stable enough to withstand stringent purification.

**Figure 2.**
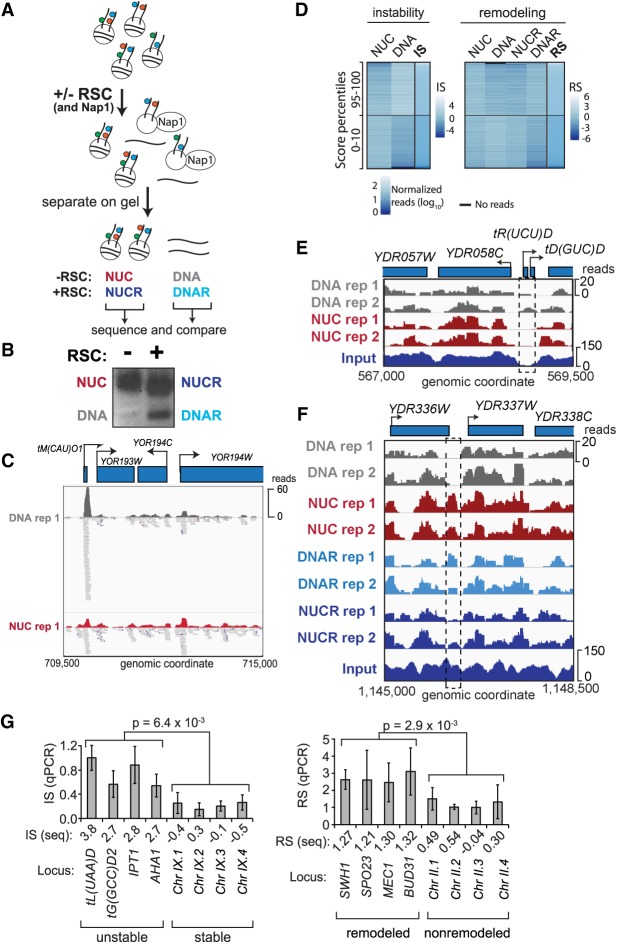
Chromatin remodeling and instability studies performed on native chromatin. (*A*) Schematic of the nucleosome disassembly assay. (*B*) Nucleosomes incubated +/− RSC were separated by native agarose gel electrophoresis. The four indicated bands were excised and the DNA extracted and sequenced. (*C*) Representative example of highly unstable nucleosome at tM(CAU)O1 (Chromosome XV), enriched in the ‘DNA’ band after incubation but also detected in ‘NUC.’ (*D*) Heat maps of read densities for nucleosomes with different IS and RS, respectively, comparing distinct percentiles. *Left* boxes, distribution of read densities in bands NUC and DNA for corresponding IS distributions. *Right* box, equivalent distribution for bands used to calculate RS; 10,000 randomly sampled windows in the 10th and 95th percentiles are shown. (*E*) Region encompassing a representative unstable nucleosome (dashed box; Chromosome IV [IS = 1.0]). (*F*) As *E*, but for a strongly remodeled nucleosome (Chromosome IV [RS = 0.78]). (*G*) qPCR validation, using primers for the indicated regions. *Left*, plot of instability index for qPCR (IS-qPCR). Four unstable nucleosomes followed by four stable nucleosomes. The *P*-value was calculated using the Wilcoxon *t*-test; error bars show standard deviations from four biological replicates. *Right*, as *left*, but for remodeling. Three biological replicates were performed.

A number of assays for measuring chromatin remodeling in vitro have been developed. We chose a simple disassembly assay, which involves incubating the nucleosome library with ATP and the histone chaperone Nap1, with or without RSC (Lorch et al. 2006). In this assay, RSC binds to nucleosomes and transfers the histones to Nap1, thereby releasing ‘naked’ DNA (Fig. 2A). Under certain conditions, reaction intermediates can be observed (tetramers or hexasomes (Lorch et al. 2006; Kuryan et al. 2012), but for simplicity, we chose to compare the input nucleosomes with the final naked DNA product. While it represents a further simplification, the general term ‘remodeling’ is hereafter used to describe the successful RSC-dependent release of such products. To separate the ejected DNA product from the nonremodeled nucleosomes, the reactions were subjected to native agarose gel electrophoresis (Fig. 2B) and DNA of the four bands isolated by gel-extraction. The upper bands, harboring nucleosomes, were named NUC (no RSC) and NUCR (with RSC), whereas the lower, ‘naked’ DNA bands were named DNA (no RSC) and DNAR (with RSC). A set of control reactions for the RSC-dependent reaction confirmed that the assay was indeed dependent on Nap1, ATP, and RSC (Supplemental Fig. S2A). Over limiting time, RSC remodels only a subset of the input nucleosomes (Supplemental Fig. S2B, left). Although we here confined our analysis to mononucleosomes, the reaction also worked on dinucleosomes (Supplemental Fig. S2B, right). The extracted DNA was sequenced after paired-end adapter ligation, enabling us to map each nucleosome to the reference yeast genome. Fragments from all four bands had the length expected for nucleosomal DNA containing a short linker (Supplemental Fig. S2C). Indeed, the mean length of nucleosome fragments was similar between the four different categories (NUC, NUCR, DNA, DNAR) and across the different stability groups defined below (Supplemental Fig. S2D). Even after experimentation and gel-extraction of the underlying DNA (Fig. 2A,B), the majority of the genome was still detected by deep sequencing of the DNA libraries: Over 83% of the genome was thus covered by at least five reads in DNA + NUC (and >81% in DNAR + NUCR) in at least one of the experimental replicates, and both replicates again showed the typical nucleosomal pattern (Supplemental Fig. S2E). These patterns are highly similar to those previously reported by Kaplan et al. (2009), also using MNase digestion. The pile-ups of reads across bands after incubation showed that these indeed came from the same nucleosomes (compare pile-ups of reads in DNA and NUC in the example shown in Fig. 2C).

Nucleosome position calling from MNase-derived sequencing data is very dependent on the specific software, so we chose a window-based approach as a robust method for quantifying nucleosome occupancy. For this purpose, we divided the yeast genome into 167-bp sliding windows with a step size of 25 bp. As previously reported for MNase-seq for nucleosomal DNA (Chung et al. 2010), we observed some enrichment for GC-rich reads and therefore normalized the read counts for the GC content for each.

In order to quantify the stability of each nucleosome, we calculated the normalized log-ratio of reads between the DNA and NUC samples per window using both replicates. The resulting Instability Score (IS = log_2_[DNA/NUC]) measures the relative stability of nucleosomes across the genome, with high scores suggesting the presence of unstable nucleosomes. Similarly, we quantified the degree of RSC-dependent nucleosome disassembly by calculating the log-ratio of reads from the DNAR and NUCR samples, before subtracting the Instability Score to account for instability. The resulting Remodeling Score (RS = log_2_[DNAR/NUCR] – IS) measures the effect of RSC-dependent remodeling, with higher scores reflecting a more strongly remodeled nucleosome. We divided the sets in eight percentiles (0%–10%, 10%–25%, 25%–50%, 50%–75%, 75%–90%, 90%–95%, 95%–99%, and 99%–100%). Finally, overlapping or adjacent windows in the same IS/RS-percentile were merged to obtain continuous unstable or remodeled regions. For simplicity, we will hereafter often refer simply to ‘nucleosomes,’ although these are strictly speaking merged, overlapping windows in the same IS/RS-percentile. We hereby obtained sets of 450,257 windows (corresponding to 91,431 nucleosomes) in which nucleosome instability could be measured and 437,761 windows (88,074 nucleosomes) to study RSC remodeling.

Figure 2D shows a heat map of read counts in the four bands and how they relate to the scores. The higher the IS, the more naked DNA there is compared to nucleosomal DNA (DNA/NUC). For RSC-dependent remodeling, not only must nucleosomes display higher read counts in the DNAR sample compared with NUCR, but they must also be stable in order to achieve a high RS. In general, the ISs and RSs are thus anticorrelated. It is worth noting that while the RSs can, of course, be compared among each other, their actual values do not have an intuitive meaning, and even ‘high’ RSs are frequently negative due to the normalization with the IS. For much of the remainder of the analysis, we often focus on the 99th percentile, which comprises the ∼4500 most unstable windows and the ∼4300 most remodeled windows, corresponding to ∼1600 and ∼1400 nucleosomes, respectively. Figure 2, C and E, shows a representative unstable nucleosome (stippled boxes), and Figure 2F shows a representative strongly remodeled nucleosome. For validation, we performed qPCR analyses on eight representative stable/unstable and remodeled/nonremodeled nucleosome positions (Fig. 2G).

### Unstable nucleosomes

**Figure 3.**
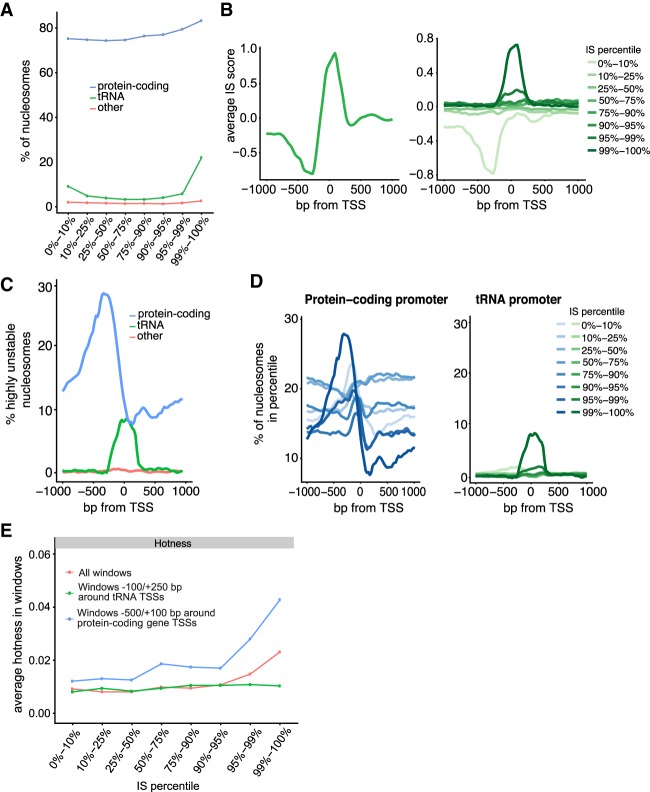
Characterization of unstable nucleosomes. (*A*) IS percentiles plotted against nucleosomes that overlap with genes. (*B*) *Left*, average ISs around the TSS of tRNA genes. *Right*, as *left*, but averages observed in different IS percentiles. (*C*) Position around the TSS of nucleosomes in the 99th IS percentile. (*D*) *Left*, as *C*, but nucleosomes in different IS percentiles around the TSS of protein-coding genes. *Right*, as *left*, but for tRNA. (*E*) Hotness of nucleosome windows in percentiles, around TSSs and across the genome.

The nucleosomes represented by the different instability percentiles were not randomly distributed across the genome. Instead, the most unstable nucleosomes were enriched in protein-coding genes, tRNA genes, and promoters compared with nucleosomes in the lower percentiles (Fig. 3A; Supplemental Fig. S3A). Unlike protein-coding genes, which are occupied by multiple nucleosomes, the short tRNA genes are typically covered by only a single nucleosome, which indeed appeared to often be highly unstable (Fig. 3B). No less than 142 of the 275 yeast tRNA genes thus contained a highly unstable nucleosome (99th percentile), and nucleosomes overlapping with tRNA gene bodies generally had a substantially higher IS compared with all others (*P*-value < 0.01, two-sided Wilcoxon rank-sum test). We also observed an enrichment of protein-coding gene promoters among nucleosomes in the higher percentiles compared with the lower percentiles (Supplemental Fig. S3B). Plotting the position of the nucleosomes in the 99th percentile, a broad area of instability was uncovered, peaking in the promoter upstream of protein-coding genes (Fig. 3C). In contrast to the peak representing tRNA genes, which was only observed with very high ISs (Fig. 3D, right), a peak in the promoter of protein-coding genes was seen in the highest percentiles but also in the lowest percentile (Fig. 3D, left), suggesting that both highly unstable and highly stable nucleosomes were detected in this area, depending on the gene. As a control, qPCR analysis showed that the release of DNA from unstable nucleosomes was indeed independent of ATP and Nap1 (Supplemental Fig. S3C), and whatever the underlying cause, instability may therefore be intrinsic to the structure of the nucleosome.

Given that many tRNA genes have a very low density of nucleosomes (Brogaard et al. 2012), we further investigated the nature of the nucleosomes called as unstable. Indeed, it even seemed possible that these were not actually all nucleosomes to start with but, for example, fragments that were cut out by MNase as naked DNA or protein–DNA complexes, which ‘contaminated’ the input material. If so, the DNA fragments cut out by MNase might be expected to differ in size from ‘true nucleosomes.’ However, the DNA reads that mapped to tRNA gene bodies were highly similar in length to those of the total pool of DNA reads (Supplemental Fig. S3D), with no statistically significant difference detected (two-sided Wilcoxon rank-sum test). Similarly, there were no differences in the lengths of DNA reads across the different stability groups, nor between DNA and NUC reads (Supplemental Fig. S2D). We also compared the nucleosomes in the different IS percentiles with a recently published nucleosome data set measured by chemical cleavage (Chereji et al. 2018) and previously by MNase digestion (Kaplan et al. 2009). Nucleosomes do appear to exist in these regions, although a decrease was observed over the windows representing the very highest ISs (Supplemental Fig. S3E,F). Inspection of nucleosome traces indicated that many of the relevant areas were often relatively nucleosome-free in the input sample, although the same regions *were* detected among the gel-purified mononucleosomes and enriched in the free DNA sample after incubation (see example in Fig. 2C).

Given these conflicting results, which might also be affected by freeze/thawing of mononucleosomes in our protocol (see Supplemental Methods), and given that we were not aiming at studying ‘nucleosome stability,’ the data sets obtained on ‘unstable nucleosomes’ were hereafter merely used as controls for experiments with RSC, to ensure that any nucleosome detected as remodeled was not merely unstable. We note, however, that one distinguishing feature of the nucleosomes classed as the most ‘unstable’ was that they were relatively enriched for ‘hot’ nucleosomes (Fig. 3E; Dion et al. 2007).

### Preferential disassembly of NFRs and TSS-nucleosomes by RSC

**Figure 4.**
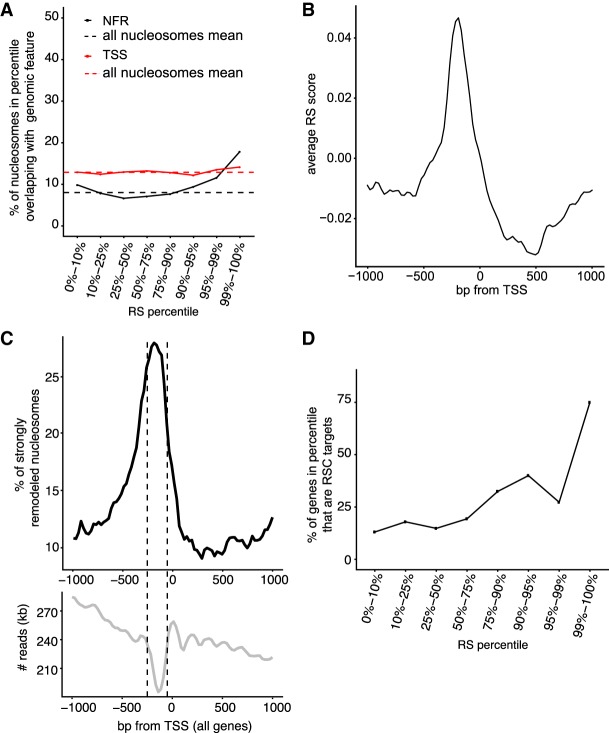
Characterization of nucleosomes remodeled by RSC. (*A*) Distribution of nucleosomes in different RS percentiles that overlap with the TSS or NFR (50–250 bp upstream of TSS). (*B*) Average RS around the TSS of genes. (*C*) *Upper*, metaprofile around the TSS of genes of strongly remodeled nucleosomes (99th percentile), relative to the general nucleosome density in the same region (*lower* graph). (*D*) RSC target genes in the different RS percentiles, relative to total number of genes in same.

Next, we investigated the characteristics of the nucleosomes that were remodeled by the RSC chromatin remodeler. As observed for unstable nucleosomes, the nucleosomes represented in the different remodeling percentiles were not randomly distributed across the genome. Indeed, the most strongly remodeled nucleosomes were relatively enriched in gene promoters and in so-called nucleosome-free regions (defined as 250–50 bp upstream of the TSS) compared with nucleosomes in lower percentiles (Fig. 4A; Supplemental Fig. S4A). In contrast to the Instability Scores, no marked difference between protein-coding and tRNA genes was observed for Remodeling Scores across this area (Supplemental Fig. S4B), so for simplicity, the analysis below is focused on mRNA and tRNA genes together (‘genes’). Analysis of the RS within 1 kb of the TSS thus showed a marked increase of the score just upstream of, and on, the TSS (Fig. 4B). These highly remodeled nucleosomes were not the same as the highly unstable nucleosomes: Indeed, only eight windows genome-wide were classified as both highly unstable *and* strongly remodeled. In fact, as mentioned above, due to the normalization of the RS by the IS, these scores are generally anticorrelated.

When we aligned the most strongly remodeled nucleosomes (99th percentile; ∼1600 nucleosomes) around the TSSs of genes, we found a broad peak upstream of the TSS, covering both the *n* − 1 and *n* + 1 nucleosome and peaking in the NFR (Fig. 4C). Of the most strongly remodeled windows, 22% were in the NFRs (indicated by dashed lines in Fig. 4C, lower), compared with 10% of all windows. As an important control, we investigated the lengths of the nucleosome fragments in the DNAR versus NUCR samples (Supplemental Fig. S2D). The lack of significant size differences between the different percentiles indicated that the highly remodeled nucleosomes do not carry additional DNA, such as adjacent promoter DNA that might have served to recruit RSC, and that the recruitment signal(s) must thus be contained within the nucleosome itself.

We conclude that RSC prefers to remodel mononucleosomes derived from promoter and TSS regions and particularly from the so-called nucleosome-free regions.

### RSC preferentially disassembles nucleosomes originating near the genes it regulates

What characterizes nucleosomes that are most strongly remodeled by RSC? GC-content did not appear to be a major defining variable, although there was a modest decrease in the higher RS percentiles (Supplemental Fig. S4C). Poly(dAdT) tracts were generally modestly enriched in remodeled nucleosomes (Supplemental Fig. S4D), in apparent agreement with the previous finding that RSC shows a preference for reconstituted nucleosomes bearing poly(dAdT) tracts (Lorch et al. 2014).

RSC harbors eight bromodomains, at least one of which has been shown to bind acetylated histone tails (Kasten et al. 2004). We failed to find one or more histone marks that were markedly enriched at higher RSs (Supplemental Fig. S4E). We failed to find a general correlation between RS and the transcription levels of the corresponding genes across the genome, as measured by RNA-seq (Nagalakshmi et al. 2008). However, we also investigated possible connections between the RS at the TSS and transcription of genes that were previously found to be dependent on RSC for their expression (Parnell et al. 2015). When the 615 genes tested by Parnell et al. were sorted into the different RS percentiles, the 122 genes affected by RSC were enriched at high RS (Fig. 4D), with more than 40% of the genes in the 90th percentile being RSC-dependent genes and RSC target genes generally having a higher RS than nontargets (*P*-value < 0.01, one-sided Wilcoxon rank-sum test). This suggests that the characteristics of chromatin at these genes are preserved to some degree in isolated mononucleosomes and, vice versa, that recognition of individual nucleosomes by RSC in vivo may indeed have significant consequences for transcription of the adjoining gene.

### RSC preferentially disassembles H2AZ-containing nucleosomes

**Figure 5.**
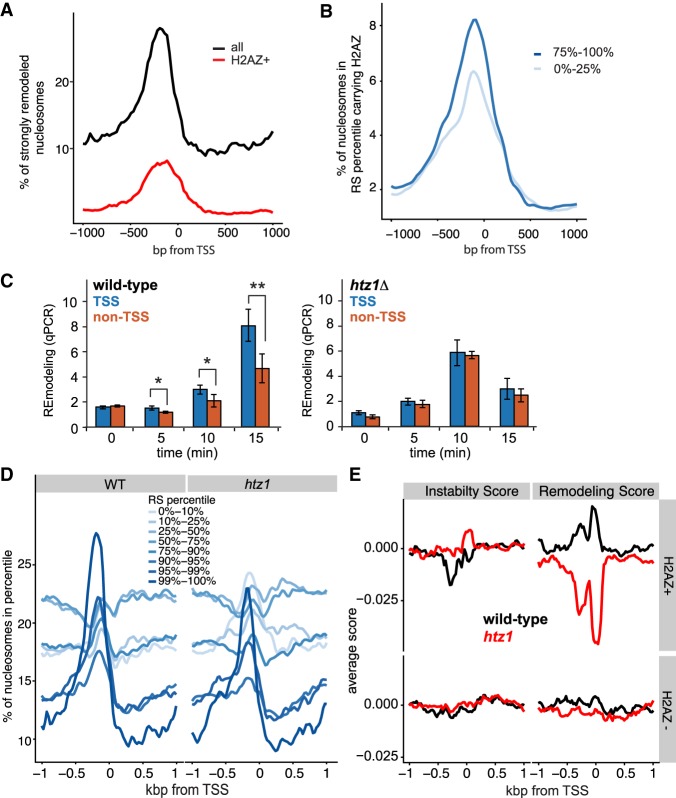
RSC prefers H2AZ-containing nucleosomes. (*A*) H2AZ-containing nucleosomes in the 99th RS percentile and their position around the TSS (red), compared with pattern for all nucleosomes in same (black). (*B*) H2AZ as in *A*, but in different RS percentiles. (*C*) Time-course of RSC remodeling preference with nucleosomes from wild type (*left*) and *htz1Δ*, (*right*) by qPCR. Blue bars show average of four strongly remodeled, H2AZ + TSS nucleosomes, and orange bars show average of four control nucleosomes (see Supplemental Fig. S5D,E). Asterisks show statistical significance; (*) *P* < 0.05, (**) *P* < 0.01; Student's *t*-test. (*D*) Position around the TSS of nucleosomes in the different RS percentiles, for wild-type (WT) and *htz1Δ* nucleosomes, respectively. (*E*) Comparison of the IS and RS for H2AZ+ and H2AZ− around the TSS of genes. For ease of comparison, the genomic mean of each score was set to zero by subtracting the genome-wide mean from each base pair.

DNA sequence and histone marks provided somewhat limited information about the mechanism underlying the nucleosome preference of RSC. However, another candidate feature is the histone variant H2AZ, which is enriched around the TSS of genes (Albert et al. 2007), in a profile which is very similar to that of the highly remodeled nucleosomes (cf. Supplemental Fig. S5A and Fig. 4B). We therefore looked specifically at nucleosomes containing H2AZ. For this purpose, we categorized nucleosomes as H2AZ+ if they were in the top 30th percentile of H2AZ levels (Albert et al. 2007). Many of the strongly remodeled nucleosomes making up the peak just upstream of the TSS indeed carried H2AZ (Fig. 5A). Moreover, the proportion of promoter regions carrying H2AZ increased with the RS of those regions (Fig. 5B). Promoters that carry H2AZ have been suggested to preferentially be occupied by nucleosomes that are rapidly exchanged in vivo (‘hot’ nucleosomes) (Dion et al. 2007). However, the RS was not correlated with ‘hot’ nucleosomes near the 5′ end of genes (Supplemental Fig. S5B), in contrast to the IS (see Fig. 3E). This indicates that the efficient RSC-mediated remodeling of H2AZ-containing nucleosomes observed in vitro is not due to such nucleosomes naturally being exchanged rapidly.

In order to experimentally address a putative causative role for H2AZ in the preference of RSC for TSS nucleosomes, we performed remodeling assays using nucleosomes prepared from *htz1Δ* cells (Supplemental Fig. S5C) and compared the resulting preference with that observed for wild-type nucleosomes. Initially, we selected four individual nucleosomes that we had found to be strongly remodeled and which carry H2AZ (Albert et al. 2007). These were compared with four control nucleosomes (Supplemental Fig. S5D). Using qPCR to detect remodeling in these regions, we found that RSC indeed showed a preference for H2AZ-containing nucleosomes at the TSS when presented with nucleosomes from wild-type cells but not with nucleosomes from *htz1*Δ cells, even though *htz1*Δ nucleosomes were, of course, still remodeled due to the general ‘background’ activity of RSC (Fig. 5C; Supplemental Fig. S5E).

We also prepared DNA libraries from experiments with *htz1Δ* nucleosomes for deep sequencing and analyzed the resulting data in the same manner as before, computing Instability and Remodeling Scores genome-wide. Parameters such as genome coverage (over 82% of the genome were covered by at least five reads in DNA + NUC and >87% in DNAR + NUCR in at least one of the experimental replicates), nucleosome positioning, nucleosome fragment lengths, and the distribution of raw DNA and NUC reads to windows in the different IS percentiles were similar to those observed in wild type, indicating that the data sets were suitable for comparison (Supplemental Figs. S2C, S6A,B). The nucleosome occupancy metaprofiles in the different RS percentiles were similar between WT and *htz1Δ* nucleosomes around the TSS of genes, with some notable exceptions: The large peak of the 99th percentile in WT was much reduced with *htz1Δ* nucleosomes and the 95th percentile peak was largely absent (Fig. 5D). This was accompanied by the 10th percentile for *htz1Δ* showing a peak of the same height as that of the 99th percentile. Indeed, only 4% of the strongly remodeled windows (99th percentile) in WT were also detected in the 90th percentile RS with *htz1Δ* nucleosomes, and a subgroup from the 99th percentile in WT of four times the size (16%) moved to the bottom 10th percentile of the *htz1Δ* RS. This indicates an underlying reordering of the preferred nucleosome remodeling positions in *htz1*Δ cells.

To further analyze these data, we again divided the genome into 25-bp bins (the step size of the windows) and computed the average RS. We then divided all bins into H2AZ+ (as above; top 30th percentile H2AZ density) (Albert et al. 2007) and H2AZ− (bottom 30th percentile) and compared their IS and RS around the TSS of genes (Fig. 5E). While the metaprofiles for the IS and RS for H2AZ− windows were similar (lower panels), the metaprofiles of the RS of the H2AZ+ windows displayed a stark difference between WT and *htz1Δ* (upper panel on the right): The WT RS thus increased on the TSS, while the opposite was observed with the *htz1Δ* RS, which reached a minimum in the same area. The ISs for the same regions were similar and so cannot account for this difference (Fig. 5E, upper panel on the left). We conclude that RSC prefers to remodel mononucleosomes originating from around the TSS in genes and that this preference is to a significant degree mediated via H2AZ.

**Figure 6.**
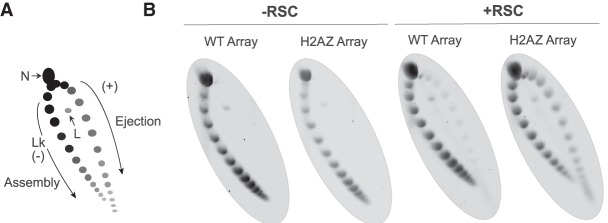
Reconstituted H2AZ-containing nucleosome arrays are preferential RSC targets as well. (*A*) Schematic of the principle of the nucleosome array ejection assay, with supercoiled plasmid (topoisomer) distribution revealed by 2D gel. (Lk) Linking number, (N) nicked, (L) linear. RSC-dependent changes in this assay are ATP-dependent (Clapier et al. 2016). (*B*) Nucleosome arrays assembled with canonical octamers (WT Array) or with H2AZ-containing octamers (H2AZ Array), incubated +/– RSC. A representative replicate is shown.

To finally investigate nucleosome remodeling by RSC using an independent, complementary system, we employed nucleosome arrays that were assembled on circular DNA plasmids using recombinant histone proteins in the presence of DNA topoisomerase I (Clapier et al. 2016). Nucleosome ejection by RSC alone from such plasmids causes a change in topoisomer distribution, with progressively less assembled states (and successively fewer nucleosomes) distributed in a clockwise manner along an arc, and any unassembled plasmids present at the lower right terminus (Fig. 6A). Arrays assembled with either H2A- or H2AZ-containing nucleosomes showed similar topoisomer distribution (Fig. 6B, left). The H2AZ arrays were superior substrates for RSC compared arrays assembled with canonical histone H2A (Fig. 6B, right), consistent with the idea that RSC is more efficient at ejection of H2AZ nucleosomes.

These results strongly support the finding that RSC prefers nucleosomes containing H2AZ.

## Discussion

The challenge of understanding the processes that take place in natural, eukaryotic chromatin, particularly at the molecular or biochemical level, is substantial. Reconstituting such chromatin with purified components in vitro is extremely challenging, and the study of biochemical mechanisms in vivo is very difficult as well. Here, we present a new molecular tool which we believe can help fill the gap between these approaches: a ‘library’ of mononucleosomes, which can be used to study different biochemical characteristics of natural chromatin as well as the substrate preference of chromatin binding factors, ATP-dependent remodelers, and chromatin-modifying enzymes. The validity of the approach is suggested by the results themselves: The finding that RSC-remodeled nucleosomes have specific and highly sensible characteristics (i.e., genome position, underlying DNA sequence, and H2AZ content, for example) indicates that the methodology works. One might have feared, for example, that random nucleosome regions had resulted from these experiments. Instead, the nucleosome-selecting factor tested—the chromatin remodeler RSC—does indeed make meaningful choices from this library, which helps further the understanding of the biology of both nucleosomes, chromatin, and RSC.

What specifies a particular genomic location—whether it is a promoter, a TSS, a telomere, or a centromere? In many cases, there are conserved DNA sequence motifs, as is the case with telomeres and recognition-sites for DNA-binding transcription factors, for example. But what about when there are no conserved sequence elements, as is the case for many promoters and TSSs? One obvious possibility is that it is exclusively through the combined presence of nearby protein-binding sites that a genomic locus comes to acquire its functional identity. According to that model, the spatial context in which a region exists is crucial for that area to ‘know’ its function. Some evidence for this idea has grown with the advent of techniques for mapping long-range interactions on chromatin (de Wit and de Laat 2012; Denker and de Laat 2016). However, even though distant interactions affect local function, it is not likely that they are sufficient to establish local identity. For one, exclusive dependence on distal regions would severely restrict co-evolution of functionally linked areas. It would also fail to explain how regions of short length can often be cloned into another genomic locus, while apparently remaining fully functional. It thus seems likely that loci also carry information about their identity on a local scale. The results of this study indicate that, actually, a large amount of information about regional genomic function may be inherent, contained in single nucleosomes, and independent of the surrounding context, in apparent agreement with recent data that argue for the absence of an organized, higher­order chromatin structure (Ou et al. 2017). This applies to at least three classes of nucleosomes: unstable nucleosomes on promoters of protein-coding genes and on tRNA genes, and nucleosomes near the TSS of genes, the latter of which were found to be preferential targets of RSC. Identity is preserved in the individual nucleosome, free from the necessity for any higher-order chromatin structure: When isolated and incubated in vitro, these mononucleosomes contain sufficient information to continue to at least partially function as if they were still embedded in their natural genomic context.

Previous work has shown that RSC plays a crucial role in establishing NFRs in vivo (Hartley and Madhani 2009) and in vitro (Wippo et al. 2011). Using mononucleosomes purified from cells, we now find that RSC preferentially remodels the individual mononucleosomes on the TSSs and promoter regions, and particularly those within the NFR. This complements, generalizes, and extends the discovery that RSC intrinsically prefers the promoter nucleosomes on a purified *PHO5* chromatin circle (Lorch et al. 2011) and that, in vivo*,* RSC associates most strongly with the first three genic nucleosomes (Yen et al. 2012). Our data indicate that much of the information required for this preference must be carried by the selected nucleosomes themselves, without the need for a more complex promoter structure, including nearby transcription factor-binding sites. Moreover, the choice made by RSC among isolated mononucleosomes in vitro is indeed highly relevant to transcription in living cells, as it preferentially recognizes and remodels mononucleosomes that originate from the genes it regulates in vivo.

We also find that the preference of RSC for these nucleosomes correlates with the presence of H2AZ and that H2AZ is indeed required to help establish this preference. A role for H2AZ in stimulating chromatin remodeling has been shown for the ISWI family of chromatin remodelers (Goldman et al. 2010), but it remains to be investigated how H2AZ facilitates RSC action and whether it does so by recruitment, catalytic stimulation, or by alternative means. We note that previous experiments on chromatin assembled in vitro showed that a mononucleosome containing H2AZ was less readily remodeled than a canonical nucleosome, not only by RSC but also by other chromatin remodelers tested (Li et al. 2005). One possible explanation for the discrepancy between the outcome of these previous experiments and our results is that the reconstitution experiments by Li et al. were performed with DNA containing a single, strong nucleosome positioning sequence, which might affect chromatin remodeling in a manner distinct from that used in our study, which used either natural mononucleosomes or a closed circular plasmid containing recombinant nucleosome arrays. Future work will determine whether the observed, preferential remodeling of H2AZ nucleosome arrays is due to nucleosome ejection, a major structural alteration, or a combination thereof.

Somewhat against our expectation, we failed to observe a significant correlation of RSC activity with a specific histone modification, such as acetylation. This is in contrast to prior evidence, which showed that acetylation *does* impact RSC function (see, for example, Chatterjee et al. 2011; Lorch et al. 2011). Indeed, Bartholomew and coworkers reported that histone H3 tail acetylation enhanced RSC recruitment and that it also increased nucleosome mobilization and H2A/H2B displacement in a bromodomain-dependent manner (Chatterjee et al. 2011). However, while histone acetylation stimulated recruitment of RSC and nucleosome remodeling via both octamer sliding and hexasome formation, it did not markedly stimulate histone eviction/ejection. Thus, rather than contradicting previous findings, our failure to detect a significant effect of histone acetylation might simply be due to the specific histone eviction assay chosen for our study. It is, however, also worth pointing out that having eight bromodomains might provide RSC with remarkable flexibility in detecting and using many different acetylation marks/positions. Therefore, one would not necessarily expect a large reliance on a *single* mark but possibly rather a general preference for highly modified nucleosomes. Indeed, such nucleosomes are generally found in promoters and around the TSS, which are also the regions preferred by RSC in our study.

In conclusion, the experimental system presented here provides a new tool for studying chromatin in vitro in a manner that preserves the natural sequences and epigenetic marks. Its chief advantages are that (1) the entire genome can be interrogated simultaneously, (2) the ‘indirect’ influences from processes occurring on chromatin in vivo have been removed, and (3) nucleosomes harbor most, if not all, the epigenetic features as they are found inside the cell. While the experimental system was established based on a yeast nucleosome library and using RSC as an example of the ‘nucleosome selectivity factor,’ it should be possible to similarly apply it to nucleosomes and chromatin-associated factors from other cell types.

## Methods

### Purification of yeast genomic chromatin

Nucleosomes were prepared from strain W303 (wild type) or isogenic *htz1Δ*. Nuclei were prepared largely as described in Almer and Hörz (1986). Nucleosomes were prepared from nuclei by digestion with MNase (New England Biolabs); these were subjected to DEAE chromatography and then loaded on a staggered 20%–45% sucrose gradient. Fractions containing the final, purified mononucleosomes were pooled.

### RSC-dependent nucleosome disassembly assay

The assay was adapted from the protocol described in Lorch et al. (2006). RSC: Nucleosome molar ratio was 1:4–1:2. After analysis, gel slices were excised and DNA extracted using a commercial kit (Life Technologies GeneJET). This DNA was used for sequencing or qPCR analysis. The nucleosome array ejection assay is described in Clapier et al. (2016); the RSC:nucleosome molar ratio was 1:2 and incubation was for 90 min.

### High-throughput sequencing

Adapters were ligated to mononucleosomal DNA using the TruSeq ChIP-seq Sample Prep (Illumina), and sequenced on Illumina HiSeq 2500.

### Nucleosome analysis

Relative recoveries of individual sequences in the four bands (NUC, DNA, NUCR, and DNAR) were determined by standard qPCR using the DNA obtained from a nucleosome disassembly assay. Native chromatin immunoprecipitation was performed using Protein A Dynabeads (Life Technologies); DNA was purified using a commercial PCR purification kit (Life Technologies GeneJET). Antibodies used were all from Abcam: #8580 (H3K4me3), #9050 (H3K36me3), and #1791 (H3). Primers for *YEF3* and *SSP120* were designed according to Kim and Buratowski (2009).

### Protein purification

TAP-tagged RSC was purified from Rsc2-TAP cells according to Lorch and Kornberg (2004). His-tagged Nap1 was purified from *Escherichia coli* according to Hizume et al. (2013).

### Bioinformatic analysis

Complementary paired-end reads were merged using FLASH (Magoc and Salzberg 2011) and then as single end reads aligned to the sacCer3 genome using Bowtie 2 (Langmead and Salzberg 2012). Read counts per sliding window were normalized for GC content with the R package EDASeq (Risso et al. 2011). Windows with fewer than five reads in DNA + NUC or DNAR + NUCR in both replicates were excluded. Normalized log-fold changes in the two replicates between the read counts in DNA and NUC (DNAR and NUCR) per window were computed with the R package DESeq2 (Love et al. 2014). All gene start sites (TSSs) were taken from Ensembl release 91 (Zerbino et al. 2018). GC-contents were determined from the nucleosome sequence in the reference genome. Poly(dAdT) tracts were defined as at least five consecutive As or Ts in a sequence and computed in a similar manner. Histone mark data, H2AZ scores, and hotness were obtained from Pokholok et al. (2005), Albert et al. (2007), and Dion et al. (2007), respectively. Nucleosome occupancy data were obtained from Kaplan et al. (2009), Brogaard et al. (2012), and Chereji et al. (2018). Gene expression data (RNA-seq) was obtained from Nagalakshmi et al. (2008). We used HybMap expression data from Parnell et al. (2015) to define RSC-target genes.

Further details on all the methods briefly outlined above can be found in Supplemental Materials.

## Data access

All raw and processed sequencing data from this study have been submitted to the ArrayExpress database at EMBL-EBI (www.ebi.ac.uk/arrayexpress) under accession number E-MTAB-7926.

## Supplementary Material

Supplemental Material
