# Supplementary material for: Genome-wide reconstitution of chromatin transactions reveals that RSC preferentially disrupts H2AZ-containing nucleosomes
Source: Genome Res. 2019 Jun;29(6):988–98. doi: 10.1101/gr.243139.118 (PMC6581049; doi:10.1101/gr.243139.118)
Supplement: Supplemental Material [file supp_29_6_988__index.html]

Genome-wide reconstitution of chromatin transactions reveals that RSC preferentially disrupts H2AZ-containing nucleosomes — Supplemental Material 

# Genome-wide reconstitution of chromatin transactions reveals that RSC preferentially disrupts H2AZ-containing nucleosomes

## Supplemental Material

- Supplementary\_Materials\_and\_Methods-FINAL.docx
- Supplemental\_Figures\_ALL.pdf
