# Supplementary material for: Genome-wide reconstitution of chromatin transactions reveals that RSC preferentially disrupts H2AZ-containing nucleosomes
Source: Genome Res. 2019 Jun;29(6):988–98. doi: 10.1101/gr.243139.118 (PMC6581049; doi:10.1101/gr.243139.118)
Supplement: Supplemental Material [file supp_gr.243139.118_Supplementary_Materials_and_Methods-FINAL.docx]

**SUPPLEMENTARY MATERIALS AND METHODS**

**Purification of yeast genomic chromatin**

Nuclei were prepared by an adaptation of the protocol described in (Almer and Horz 1986). Six litres of YPD were inoculated with 300 ml saturated starter culture in the morning to harvest cells grown to mid-log phase (4-6 cells/ml) in the early afternoon. Cells were pelleted at 3,500 x g for 10 minutes and resuspended in 240 ml (50 mM Tris pH 7.5, 40 mM DTT) for gentle shaking at 30˚C for 15 minutes. Cells were pelleted at 11,000 x g for 5 minutes (JA-10 rotor) and resuspended in 60 ml 0.5x YPD/1 M sorbitol at room temperature (from here on all buffers were supplemented with standard protease inhibitors and 5 mM sodium butyrate and 5 mM trichostatin A as deacetylase inhibitors). For spheroblasting, 100 mg of zymolase 20T were added, and the cells shaken slowly for 30-50 minutes at 30˚C, until the OD_600_ of a 1:100 dilution in 1% SDS was less than 5% of the initial OD_600_ (e.g. 0.034 vs. initial 0.330). It is important not to overdigest, since the histone tails are sensitive to proteolysis. Zymolysis was stopped by addition of 100 ml cold 1 M sorbitol, and all subsequent operations were performed in the cold. Spheroblasts were pelleted and rinsed twice by centrifuging at 1,500 x g for 5 minutes (JA-10 rotor) and washing twice with 200 ml cold 1 M sorbitol. The rinsed spheroblasts were resuspended in 100 ml Ficoll Buffer (18% Ficoll 400, 20 mM potassium phosphate pH 6.8, 1 mM MgCl_2_, 0.25 mM EDTA, 0.25 mM EGTA) to lyze cells and release the nuclei. Nuclei were centrifuged at 45,000 x g for 20 minutes (Ti-45 rotor), then resuspended in 30 ml SucB1 (0.34 M sucrose, 20 mM Tris pH 7.5, 50 mM KCl, 5 mM MgCl_2_). The resuspended nuclei were then layered on 15 ml SucB2 (1.7 M sucrose, 20 mM Tris pH 7.5, 50 mM KCl, 5 mM MgCl_2_) and centrifuged at 115,000 x g for 30 minutes (SW-32 rotor) to further clean up the nuclei. These were resuspended in 10 ml SucB1, aliquoted to ~12 x 1 ml, and centrifuged at 18,000 x g for 1 minute. Supernatants were discarded, and pelleted nuclei frozen in liquid nitrogen for storage. Frozen nuclei could be stored indefinitely at -80˚C.

Nucleosomes were prepared from nuclei over two days. On the morning of day 1, one aliquot of nuclei was thawed and resuspended in 500 µl cold MNase Buffer (50 mM NaCl, 13 mM Tris pH 8.0, 6.4 mM CaCl_2_, 0.2 mM EDTA, 0.2 mM EGTA), before adding 10 µl 0.1 M CaCl_2_ and protease and deacetylase inhibitors as above. Resuspended nuclei were pre-heated for 90 seconds at 37˚C, and digestion performed with ~40,000 U MNase (~20 µl of 2,000 U/µl from New England BioLabs) for 2 minutes at 37˚C. Digestion was stopped by addition of 25 µl 0.5 M EGTA and placing of the tube on ice for 2 minutes. Optimal MNase concentrations were determined in a trial digestion for each batch of nuclei, by testing five different concentrations of MNase with 100 µl resuspended nuclei and choosing the concentration that yields mostly mono-nucleosomes, but still results in formation of some oligo-nucleosomes. It is crucial that the digestion be performed as fast as possible, and that all other steps be performed on ice or in the cold room to avoid proteolysis of histone tails. After digestion, the incubated nuclei were spiked with more protease inhibitors, then centrifuged at 18,000 x g for 1 minute. The supernatant containing mono-nucleosomes was kept and centrifuged one more time to remove remaining nuclei. The solution should be brown to yellow in colour, and clear. For DEAE chromatography, we used a self-poured 1.2 cm x 7 cm column (6 ml bed volume) equilibrated in DEAE-NucB-20 (20 mM NaCl, 10 mM Tris pH 7.5, 1.5 mM MgCl_2_, 10% glycerol). All 500 µl of nuclear lysate were loaded at 0.3 ml/min, and elution was performed in a 20-800 mM NaCl gradient in DEAE-NucB at 0.2 ml/min over 40 minutes. Chromatin eluted at about 400 mM in the second half of the major peak (See Figure 1B). The high salt concentration of ~400 mM used for elution from DEAE ensured that DNA-binding transcription factors were released and washed off, while the gradient centrifugation served to remove contaminants and separate free nucleosomes from nucleosomes bound by other proteins. Before proceeding with the sucrose gradient, fractions containing chromatin were identified with a quick agarose gel analysis by extracting DNA from 20 µl using the Qiagen PCR purification kit, performing a brief digestion with RNAse A and separating bands on a 1.5% agarose gel. Fractions containing chromatin were pooled and loaded on a staggered 20-45% sucrose gradient consisting of 5 ml each of 45, 40, 35, 30, 25 and 20% sucrose in Sucrose Buffer (30 mM NaCl, 10 mM Tris pH 7.5, 1 mM EDTA, 1 mM EGTA). Ultracentrifugation was performed at 200,000 x g for 26 hours (SW41 rotor). Fractions of ~500 µl were then collected by piercing the bottom of the tube (~8 drops per fraction, ~20 fractions total). To identify the fractions containing DNA, 15 µl of each fraction was incubated with ~30 µg RNAse A for 20 minutes at 37˚C, before adding 3 µl loading buffer containing 1% SDS, and separating bands on a 1.5% agarose gel. Fractions containing mono-nucleosomes were pooled, distributed into 25 or 50 µl aliquots, flash-frozen in liquid nitrogen, and stored at -80˚C. Final recoveries were ~1 to 1.5 ml at concentrations of ~20 ng/µl, corresponding to 20-30 µg of DNA. Each aliquot was thawed only once. We recommend testing the integrity of the histone tails by performing a western blot for histone H3 and checking for the absence of a lower band corresponding to proteolyzed histone. DNA concentrations in the final material were determined by qPCR using a standard curve of two different PCR products of known concentration that covered a part of the genome (primers P764/765 and P788/789).

**Strains used in this study**

Nucleosomes were prepared from strains W303 (wild-type) and *htz1∆* from the Saccharomyces Genome Deletion Project (Brachmann et al. 1998).

**Protein purification**

TAP-tagged RSC was purified from Rsc2-TAP cells according to (Lorch and Kornberg 2004). His-tagged Nap1 was purified from *Escherichia coli* according to (Hizume et al. 2013).

**RSC-dependent nucleosome disassembly assay**

The assay was adapted from the protocol described in (Lorch et al. 2006), except that DNA was stained with ethidium rather than by radioactive labelling. Each reaction was performed in 20 µl volumes, with 6-10 µl nucleosomes, 800 ng RSC, 2 µg Nap1, 1 mM ATP, 20 mM potassium acetate pH 7.6, 15 mM Hepes pH 7.9, 3 mM MgCl_2_, 75 µg/ml bovine serum albumin, and protease inhibitors (Rsc:nucleosome molar ratio ~1:4 – 1:2). After incubation for 15 minutes (replicate 1) or 20 minutes (replicate 2) at 30˚C, 1 µl of ~2 mg/ml plasmid DNA was added to capture free RSC, followed by the addition of 5 µl 30% glycerol. At this point, the mixture could be flash-frozen in liquid nitrogen and stored indefinitely at -80˚C. For native gel-electrophoresis, the thawed mixture was loaded on a pre-cooled 2% agarose gel in 0.5x TBE, then run for ~30 minutes at 50 V and ~1.5 hours at 100 V. Bands were stained by soaking the gel in 1 µg/ml ethidium bromide for one hour, destained with water for 30 minutes and bands analyzed with UV light. In our experience, bands were generally very weak, with their intensity varying greatly depending on the concentration of nucleosomes. After analysis, gel slices were excised, and DNA extracted using a commercial kit (Life Technologies GeneJET), to be eluted into 50 µl elution buffer. This DNA was suitable for sequencing or qPCR analysis.

**High-throughput sequencing**

Adapters were ligated to mono-nucleosomal DNA using the TruSeq ChIP-seq Sample Prep (Illumina), amplified in 12 cycles, and sequenced in 4- or 5-plex multiplex format on Illumina HiSeq 2500.

**qPCR analysis of unstable and remodelled nucleosomes**

Relative recoveries of individual sequences in the four bands (NUC, DNA, NUCR and DNAR) were determined by standard qPCR using the DNA obtained from a nucleosome disassembly assay. Nucleosomes to investigate for analysis were chosen by scanning the coverage of all four bands for nucleosomes with the desired properties (as in Figure 2E and 2F). IS(qPCR) and RS(qPCR) are defined as IS(seq) and RS(seq), except that the log2 is not taken. Thus, IS(qPCR) = [DNA]/[NUC] and RS(qPCR) = [DNAR]/[NUCR] – IS, where the numbers in brackets are the relative concentrations of a given sequence as determined by qPCR.

**Native chromatin immunoprecipitation**

Protein A Dynabeads (25 µl, Life Technologies) were washed with PBS, pre-loaded with 4 µl antibody, then washed again twice with PBS. After resuspension in 300 µl IP-B2 (150 mM NaCl, 50 mM Tris pH 8.0, 2 mM EDTA, 0.1% NP-40 and 0.01% SDS), 50 µl nucleosomes were added. 50 µl of the input were kept for qPCR analysis, and the remaining 300 µl nutated for 3 hours at 4˚C. Nucleosome-bound beads were washed three times with 500 µl IP-B2 and DNA eluted by incubating with 50 µl Elution-B (IP-B2 with 1% SDS) for 20 minutes at room-temperature. 100 µl TE pH 8.0 were added to the eluates and to the input, and the DNA purified using a commercial PCR purification kit (Life Technologies GeneJET). Sequence quantitation by was performed by standard analysis by quantitative PCR. Antibodies used were all from Abcam: #8580 (H3K4me3), #9050 (H3K36me3) and #1791 (H3). Primers for *YEF3* and *SSP120* were designed according to (Kim and Buratowski 2009).

**Read processing and alignment**

The quality of reads was checked with FASTQC (available online at <https://www.bioinformatics.babraham.ac.uk/projects/fastqc/>). Reads were trimmed with trimgalore (parameters: --length 0) (available online at [www.bioinformatics.babraham.ac.uk/projects/trim_galore/](http://www.bioinformatics.babraham.ac.uk/projects/trim_galore/)). Complementary paired-end reads were combined into a single sequence covering the entire nucleosome using FLASH (parameters: -m 10 -M 53) (Magoc and Salzberg 2011) and then as single-end reads aligned to the sacCer3 genome using Bowtie 2 (parameters: default)(version 2.3.3.1, (Langmead and Salzberg 2012)). The resulting SAM file was converted into BAM format using SAMtools (Li et al. 2009). Duplicate reads were identified using Picard (parameters: default) (available online at <http://broadinstitute.github.io/picard/>) and subsequently removed using SAMtools. Only reads that mapped uniquely with a mapping quality of at least 30 and that were longer than 50 bp and shorter than a nucleosome with a long linker (190 bp) were retained. Reads mapping to the mitochondrial chromosome were discarded.

The final numbers of reads for the input and each band in each replicate (15 min and 20 min incubation) after trimming are shown in the table below.

|  | **Wild-type**  # reads  Replicate 1/ Replicate 2 | ***Htz1***  # reads  Replicate 1/ Replicate 2 |
| --- | --- | --- |
| **DNA** | 465,006 / 607,212 | 202,079 / 588,507 |
| **NUC** | 729,397 / 688,374 | 279,007 / 687,732 |
| **DNAR** | 377,768 / 505,395 | 360,910 / 948,949 |
| **NUCR** | 408,719 / 657,549 | 426,977 / 750,861 |
| **Input** | 6,476,151 |  |

**Sliding windows, Instability and Remodelling Scores and nucleosome regions**

We excluded both the first and last 1000 bp of each chromosome of the yeast genome. We divided the yeast genome into sliding windows of size 167bp with step size 25bp and allocated any overlapping mapped reads to the windows. As previously reported for MNase-seq for nucleosomal DNA (Chung et al. 2010), we observed an enrichment for GC-rich reads, and we therefore normalized the read counts for the GC content of that window with the R package EDASeq (Risso et al. 2011). We excluded windows with fewer than 5 reads in DNA+NUC or DNAR+NUCR in both replicates from further analysis. Read counts per window were subsequently normalized for sequencing depth and normalized log-fold changes in the two replicates between the read counts in DNA and NUC (DNAR and NUCR) per window were computed with the R package DESeq2 (Love et al. 2014). After normalization and quality filtering, 450,257 and 439,841 windows were recovered for comparison of read distributions in DNA vs NUC and DNAR vs NUCR in wild-type (449,929 and 455,986 windows for htz1 mutant respectively).

The Instability Score (IS) was defined as the log-fold change between the read counts DNA and NUC for each individual nucleosome, ie. IS = log_2_(DNA/NUC). The Remodeling Score (RS) was defined as RS = log_2_(DNAR/NUCR)-IS. We divided the sets in 8 percentiles (0-10%, 10-25%, 25-50%, 50-75%, 75-90%, 90-95%, 95-99%, 99-100%). Finally, overlapping or adjacent regions in the same percentile for IS or RS were merged to obtain unstable and remodeled regions. The median widths of these regions (median width ~ 267 for both IS and RS) are too small to contain more than one nucleosome, and we refer to these regions as nucleosomes in the remainder of the text for simplicity. To compare fragment lengths in the 4 bands (Figure S2D), we used the maximal overlapping window to assigned each read an IS/RS percentile.

**Comparison with published datasets**

The unique nucleosome map of the Widom lab (Brogaard et al. 2012) was annotated to the yeast genome sacCer3 using the R package rtracklayer (Lawrence et al. 2009). Nucleosome occupancy data from the Segal lab (Kaplan et al. 2009) was annotated to the sacCer3 version of the yeast genome using the R package rtracklayer (Lawrence et al. 2009). Newer chemical cleavage nucleosome occupancy data from the Henikoff lab (Chereji et al. 2018) was obtained and mean occupancy score per window were computed.

**Enrichment of genetic and epigenetic features**

We used the *biomaRt* package (Durinck et al. 2005; Durinck et al. 2009) to retrieve gene annotation information from Ensembl release 91 (Zerbino et al. 2018) using BioMart web services (Kasprzyk et al. 2004; Smedley et al. 2015). GC-contents were determined from the nucleosome sequence in the reference genome sacCer3 (The Bioconductor (The Bioconductor Dev Team 2014). Poly(dAdT) tracts were defined as at least 5 consecutive A’s or T’s in a sequence and computed in a similar manner. Histone mark data were obtained from (Pokholok et al. 2005). They were assigned to each window or nucleosome by first smoothing them using a rolling window of size 500 with step size 100 across the genome and then assigning their average value over a given window or nucleosome as score. H2A.Z scores and hotness were obtained from (Albert et al. 2007) and (Dion et al. 2007), respectively, then assigned to each nucleosome/window in a similar manner.

**Metaprofiles and Heatmaps**

To produce the metaprofiles of the raw reads, we used deepTools (Ramirez et al. 2016) to remove the GC bias of the raw read counts (paramters: default), and subsequently to center reads and convert the resulting bam file into a BigWig file (parameters: --minFragmentLength 140 –maxFragmentLength 190 –centerReads –binSize 1), and then to plot the metaprofiles. Other metaprofiles were built by using the regions of interest (TSSs or nucleosomes) as templates in which the presence/absence of a feature (window, nucleosome, poly(dA:dT) tracks) is noted by 1/0. When the nucleosomes were analysed in a gene context (promoter or TSS), they were first flipped accordingly to the sense of transcription. The resulting vectors were then summed and normalised by the total number of regions of interest. For the IS or RS scores, we first computed the mean scores in 25bp bins across the genome (corresponding to the step size of the windows) and subtracted the genome-wide mean. We then computed the metaprofiles the metaprofile with deeptools using the functions computeMatrix (parameters: reference-point --referencePoint TSS --beforeRegionStartLength 1000 --regionBodyLength 10 --afterRegionStartLength 1000 --missingDataAsZero --binSize 1) and plotProfile. The metaprofile plots were finally generated with the R package ggplot2 (Wickham 2016) and smoothed with a polynomial regression with a polynomial function of degree 51. The heatmaps of reads in genomic contexts were generated with the R package ggbio (Yin et al. 2012).

**Genomic features assessment**

In order to look at the genomic distribution of the nucleosome regions, we divided them into TSSs, TTSs, promoters (-600 to -100 upstream of TSS according to the sense of transcription), gene bodies and intergenic regions. The promoters were trimmed if they were overlapping a gene body upstream. We first assigned TSS (13,301 IS and 12,608 RS) and TTS (13,050 IS and 12,324 RS) or both (3,492 IS and 3,162 RS) nucleosomes; the remaining nucleosomes were intersected with promoters (16,363 IS and 15,907 RS) and then gene bodies (41,470, IS and 40,134 RS). 3,755 IS and 3,939 RS nucleosomes fall in intergenic regions.

**Gene expression analysis:**

Gene expression data was obtained from (Nagalakshmi et al. 2008) and annotated to the sacCer3 genome using the R package rtracklayer (Lawrence et al. 2009). Further, we used the genomic locations of the HybMap expression data from (Parnell et al. 2015) to compute the log-fold change of expression between the *rsc2-V457M* strain and *wild-type* for those genes in the dataset where at least half of the gene body was covered by probes. We then defined the individual expression values for each gene as the average of the (sense) probes that overlapped with the gene body. We defined genes to be RSC-targets if the absolute value of log-fold change in expression between *rsc2-V457M* strain and *wild-type* was in the 80th percentile of the absolute log-fold changes.

We assigned each of the genes a mean RS as the mean of the scores of the windows intersecting 100bp up- and downstream of the TSS of each gene respectively. This resulted in 607 genes having both a finite log-fold change and a defined RS score: 555 protein-coding, 2 tRNAs, 44 snoRNAs, 4 ncRNAs, and 1 snRNAs; of which 124 genes are RSC-targets (85 protein-coding, 33 snoRNA, 1 tRNAs, 1 snRNAs and 2 ncRNAs). We then mapped these RS values to the genome wide percentiles to define RS percentiles.

**Nucleosome Ejection Assay**

Nucleosome arrays used in the ejection assays were assembled as 100 µl reaction containing 5 µg of pBluescript plasmid DNA mixed with 23 pmol of recombinant histone octamers, containing either canonical H2A or H2A.Z (Htz1) variant, in 2 M KCl, 1x NEB4, 0.1 mg/ml BSA, in the presence of 10 U of Topoisomerase I (NEB) with a linear salt-gradient dialysis applied from 2 M to 50 mM KCl at 30°C, using an Econo-Pump (Bio-Rad) and Slide-A-Lyser Mini Dialysis units with a 7,000 molecular weight cutoff (Thermo Scientific).

Nucleosome ejection assays were performed in a 50 µl reaction by incubating 1 pmol of RSC protein complex (20nM) with 500 ng of nucleosome arrays (described above) in 10 mM Tris buffer, pH 7.4, 50 mM KCl, 3 mM MgCl_2_, 0.1 mg/ml BSA, 1 mM ATP at 30°C with shaking at 500 rpm in a Thermomixer (Eppendorf) for 90 min. Deproteinization was performed by adding 5 µl of proteinase K at 10 mg/ml and 2.5 µl of 20% SDS and incubated at 50°C for 1h. Samples were subsequently precipitated in ethanol, prior to two-dimensional separation on a 1.3% agarose gel as described in (Clapier et al. 2001). Gels were stained for 15 min in a 1 µg/ml ethidium bromide solution and scanned on a Typhoon Trio (Amersham, GE).

**References**

Albert I, Mavrich TN, Tomsho LP, Qi J, Zanton SJ, Schuster SC, Pugh BF. 2007. Translational and rotational settings of H2A.Z nucleosomes across the Saccharomyces cerevisiae genome. *Nature* **446**: 572-576.

Almer A, Horz W. 1986. Nuclease hypersensitive regions with adjacent positioned nucleosomes mark the gene boundaries of the PHO5/PHO3 locus in yeast. *Embo J* **5**: 2681-2687.

Brachmann CB, Davies A, Cost GJ, Caputo E, Li J, Hieter P, Boeke JD. 1998. Designer deletion strains derived from Saccharomyces cerevisiae S288C: a useful set of strains and plasmids for PCR-mediated gene disruption and other applications. *Yeast* **14**: 115-132.

Brogaard K, Xi L, Wang JP, Widom J. 2012. A map of nucleosome positions in yeast at base-pair resolution. *Nature* **486**: 496-501.

Chereji RV, Ramachandran S, Bryson TD, Henikoff S. 2018. Precise genome-wide mapping of single nucleosomes and linkers in vivo. *Genome biology* **19**: 19.

Chung HR, Dunkel I, Heise F, Linke C, Krobitsch S, Ehrenhofer-Murray AE, Sperling SR, Vingron M. 2010. The effect of micrococcal nuclease digestion on nucleosome positioning data. *PLoS One* **5**: e15754.

Clapier CR, Langst G, Corona DF, Becker PB, Nightingale KP. 2001. Critical role for the histone H4 N terminus in nucleosome remodeling by ISWI. *Mol Cell Biol* **21**: 875-883.

Dion MF, Kaplan T, Kim M, Buratowski S, Friedman N, Rando OJ. 2007. Dynamics of replication-independent histone turnover in budding yeast. *Science* **315**: 1405-1408.

Durinck S, Moreau Y, Kasprzyk A, Davis S, De Moor B, Brazma A, Huber W. 2005. BioMart and Bioconductor: a powerful link between biological databases and microarray data analysis. *Bioinformatics* **21**: 3439-3440.

Durinck S, Spellman PT, Birney E, Huber W. 2009. Mapping identifiers for the integration of genomic datasets with the R/Bioconductor package biomaRt. *Nature protocols* **4**: 1184-1191.

Hizume K, Yagura M, Araki H. 2013. Concerted interaction between origin recognition complex (ORC), nucleosomes and replication origin DNA ensures stable ORC-origin binding. *Genes Cells* **18**: 764-779.

Kaplan N, Moore IK, Fondufe-Mittendorf Y, Gossett AJ, Tillo D, Field Y, LeProust EM, Hughes TR, Lieb JD, Widom J et al. 2009. The DNA-encoded nucleosome organization of a eukaryotic genome. *Nature* **458**: 362-366.

Kasprzyk A, Keefe D, Smedley D, London D, Spooner W, Melsopp C, Hammond M, Rocca-Serra P, Cox T, Birney E. 2004. EnsMart: a generic system for fast and flexible access to biological data. *Genome Res* **14**: 160-169.

Kim T, Buratowski S. 2009. Dimethylation of H3K4 by Set1 recruits the Set3 histone deacetylase complex to 5' transcribed regions. *Cell* **137**: 259-272.

Langmead B, Salzberg SL. 2012. Fast gapped-read alignment with Bowtie 2. *Nature methods* **9**: 357-359.

Lawrence M, Gentleman R, Carey V. 2009. rtracklayer: an R package for interfacing with genome browsers. *Bioinformatics* **25**: 1841-1842.

Li H, Handsaker B, Wysoker A, Fennell T, Ruan J, Homer N, Marth G, Abecasis G, Durbin R, Genome Project Data Processing S. 2009. The Sequence Alignment/Map format and SAMtools. *Bioinformatics* **25**: 2078-2079.

Lorch Y, Kornberg RD. 2004. Isolation and assay of the RSC chromatin-remodeling complex from Saccharomyces cerevisiae. *Methods Enzymol* **377**: 316-322.

Lorch Y, Maier-Davis B, Kornberg RD. 2006. Chromatin remodeling by nucleosome disassembly in vitro. *Proc Natl Acad Sci U S A* **103**: 3090-3093.

Love MI, Huber W, Anders S. 2014. Moderated estimation of fold change and dispersion for RNA-seq data with DESeq2. *Genome biology* **15**: 550.

Magoc T, Salzberg SL. 2011. FLASH: fast length adjustment of short reads to improve genome assemblies. *Bioinformatics* **27**: 2957-2963.

Nagalakshmi U, Wang Z, Waern K, Shou C, Raha D, Gerstein M, Snyder M. 2008. The transcriptional landscape of the yeast genome defined by RNA sequencing. *Science* **320**: 1344-1349.

Parnell TJ, Schlichter A, Wilson BG, Cairns BR. 2015. The chromatin remodelers RSC and ISW1 display functional and chromatin-based promoter antagonism. *Elife* **4**: e06073.

Pokholok DK, Harbison CT, Levine S, Cole M, Hannett NM, Lee TI, Bell GW, Walker K, Rolfe PA, Herbolsheimer E et al. 2005. Genome-wide map of nucleosome acetylation and methylation in yeast. *Cell* **122**: 517-527.

Ramirez F, Ryan DP, Gruning B, Bhardwaj V, Kilpert F, Richter AS, Heyne S, Dundar F, Manke T. 2016. deepTools2: a next generation web server for deep-sequencing data analysis. *Nucleic Acids Res* **44**: W160-165.

Risso D, Schwartz K, Sherlock G, Dudoit S. 2011. GC-content normalization for RNA-Seq data. *BMC Bioinformatics* **12**: 480.

Smedley D Haider S Durinck S Pandini L Provero P Allen J Arnaiz O Awedh MH Baldock R Barbiera G et al. 2015. The BioMart community portal: an innovative alternative to large, centralized data repositories. *Nucleic Acids Res* **43**: W589-598.

The Bioconductor Dev Team. 2014. BSgenome.Scerevisiae.UCSC.sacCer3: Saccharomyces cerevisiae (Yeast) full genome (UCSC version sacCer3). R package version 1.4.0.

Wickham H. 2016. *ggplot2: Elegant Graphics for Data Analysis.* . Springer-Verlag New York.

Yin T, Cook D, Lawrence M. 2012. ggbio: an R package for extending the grammar of graphics for genomic data. *Genome biology* **13**: R77.

Zerbino DR, Achuthan P, Akanni W, Amode MR, Barrell D, Bhai J, Billis K, Cummins C, Gall A, Giron CG et al. 2018. Ensembl 2018. *Nucleic Acids Res* **46**: D754-D761.
