# Supplementary material for: Genome-wide reconstitution of chromatin transactions reveals that RSC preferentially disrupts H2AZ-containing nucleosomes
Source: Genome Res. 2019 Jun;29(6):988–98. doi: 10.1101/gr.243139.118 (PMC6581049; doi:10.1101/gr.243139.118)
Supplement: Supplemental Material [file supp_gr.243139.118_Supplemental_Figures_ALL.pdf]

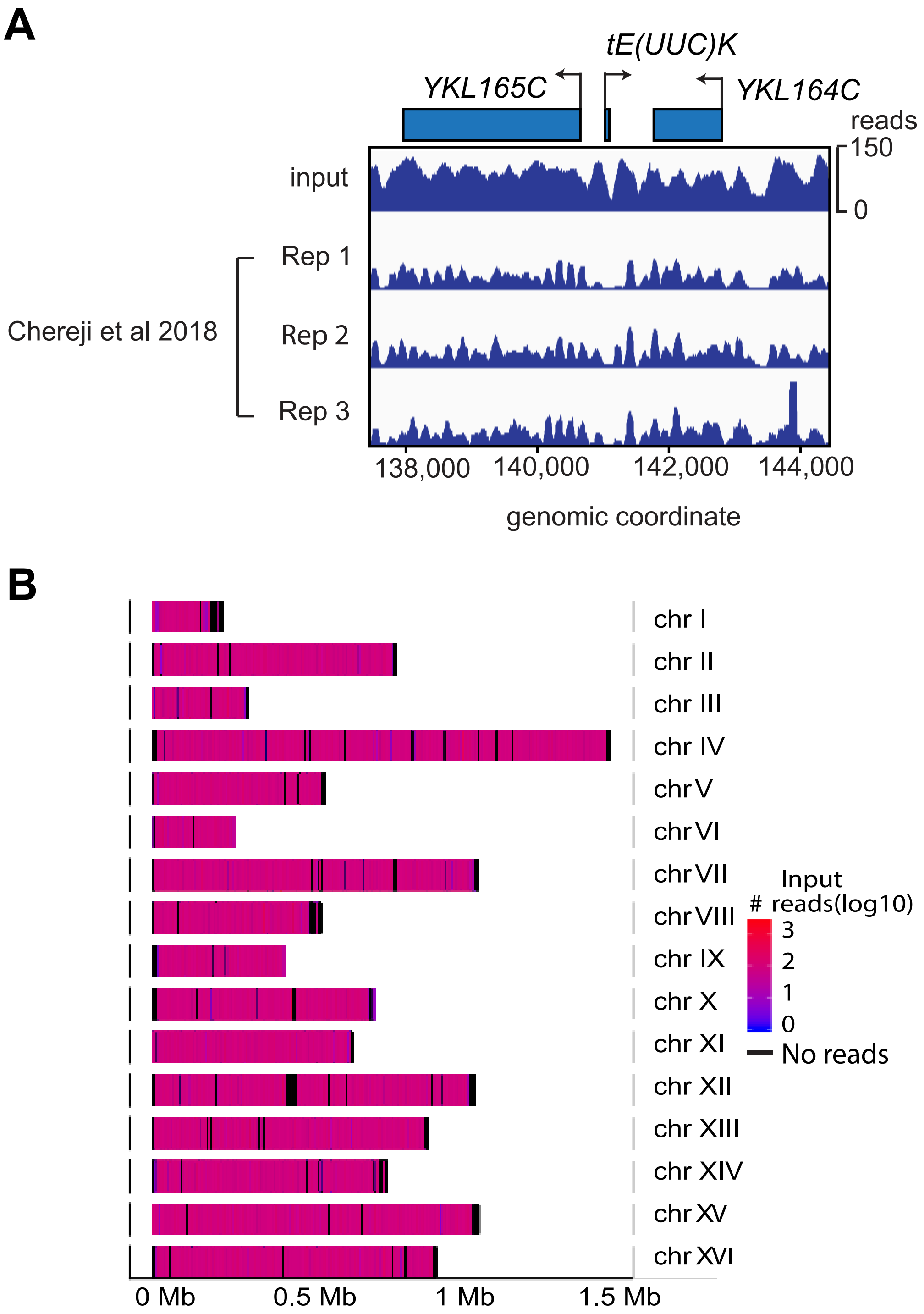

Figure S1.Nucleosome distribution. A. Raw read density in the input sample across a region on Chromosome XI, compared to the nucleosomes called by Chereji et al., 2018 based on chemical cleavage. Note that the overall patterns are very similar. B. Most of the genome is represented in the mono-nucleosome library. Read density in the input material across the different yeast chromosomes is shown.

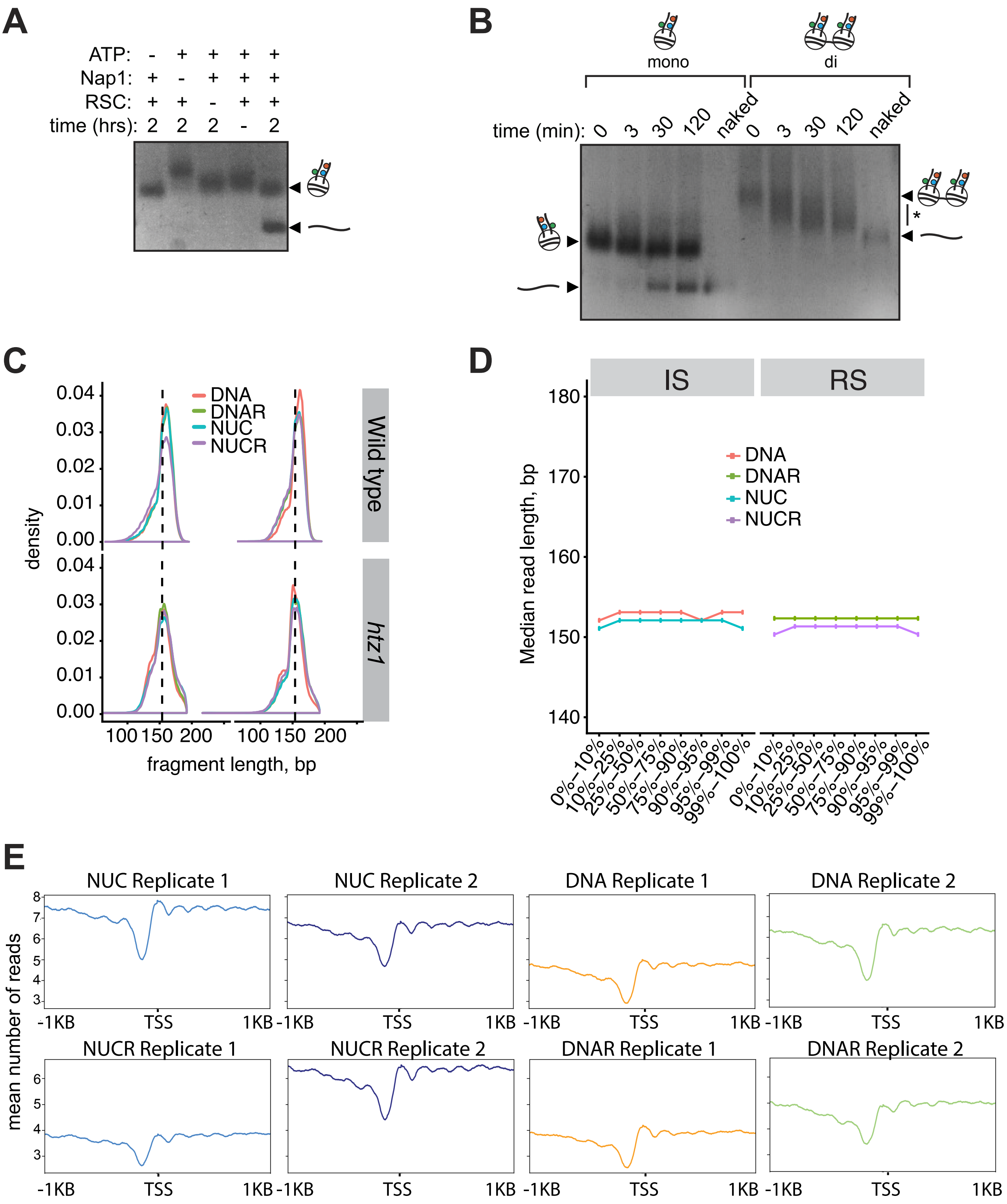

Figure S2. Characterization of assay and nucleosomes. A. Nucleosome disassembly depends on ATP, Nap1 and RSC. The assay was performed as for Figure 2B and bands separated by agarose gel-electrophoresis. B. Reaction time-course of RSC-dependent nucleosome disassembly of mono- and di-nucleosomes. Nap1 and ATP were included in excess. “Naked” shows naked DNA. Asterisk shows smear of hybrid species consisting of mono-nucleosomes and rearranged di-nucleosomes. C. DNA read lengths after trimming, merging, mapping and removal of PCR duplicates in the different categories, in nucleosome libraries from WT and htz1, respectively. Labels are as shown in Figure 2A. D. Average DNA read lengths as in C in the different IS and RS categories, respectively. IS and RS categories were assigned using the maximal overlapping window for each read. E. Average profile of mapped reads around the TSS in the different replicates is highly similar, and resembles that observed by, for example, (Kaplan et al. 2009).

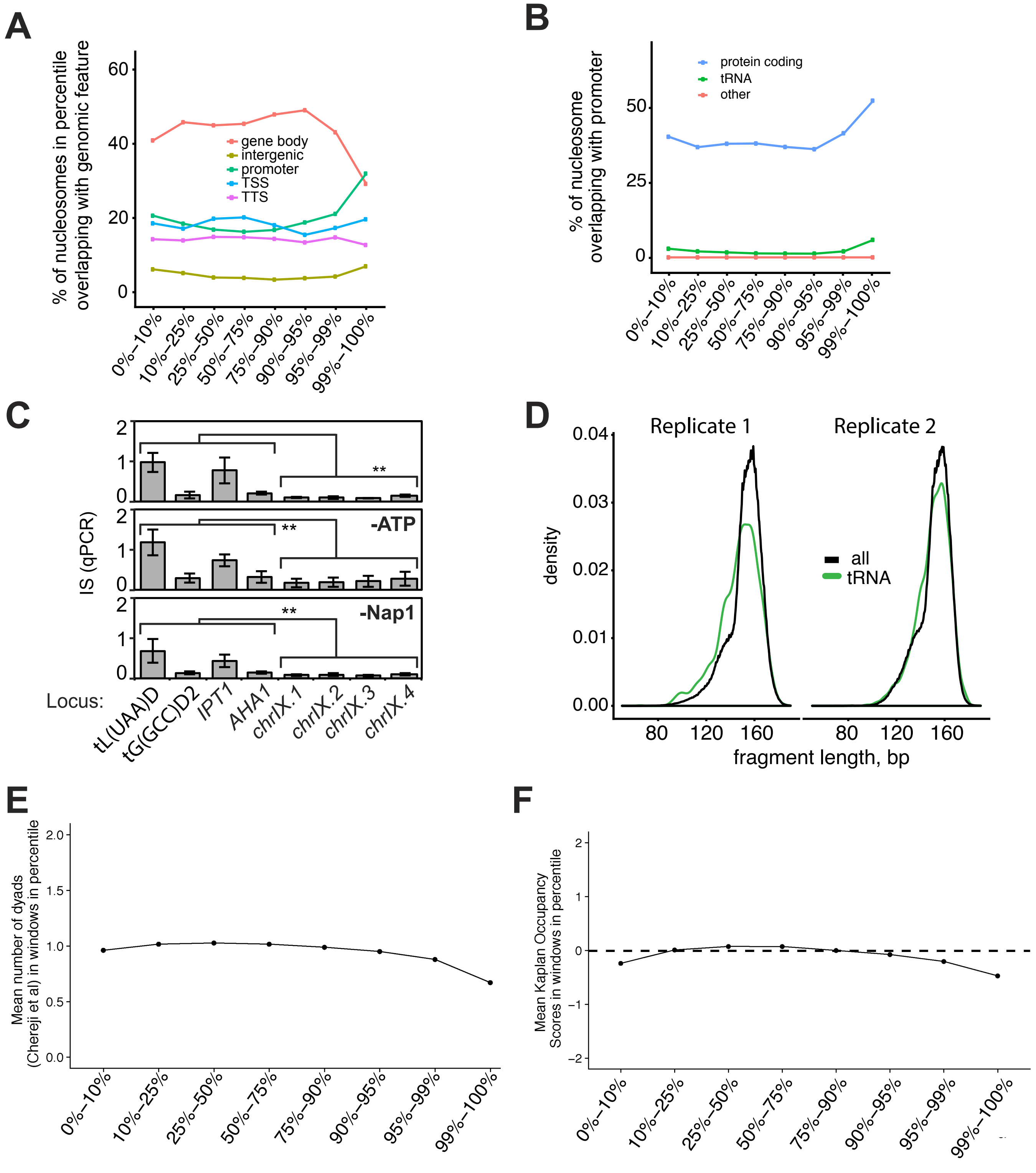

Figure S3. Characterization of intrinsically unstable nucleosomes. A. Nucleosomes that overlap with genomic features plotted against IS score percentiles, as indicated. B. Nucleosomes in the promoter region of genes plotted against IS score percentiles. C. Instability as measured in this assay does not require ATP or Nap1. Analysis as in Figure 2G. Asterisks show statistical significance by Wilcoxon t-test (**= $p < 0.01$). D. DNA read lengths at tRNA genes compared to all other reads. E-F. Number of nucleosome dyads detected by (Chereji et al. 2018)(E), and number of nucleosomes detected by (Kaplan et al. 2009)(F) across the different IS percentiles.

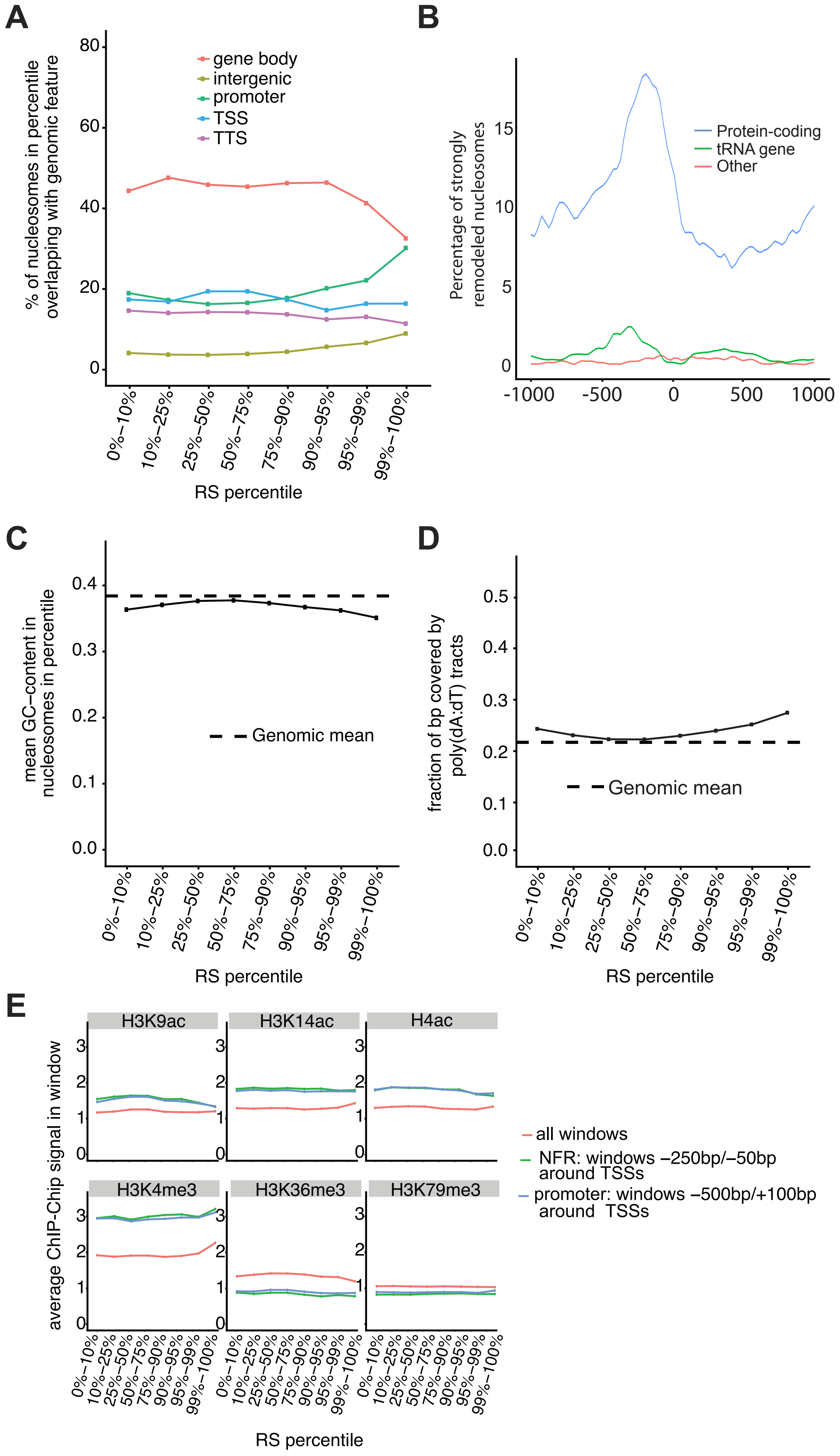

Figure S4. Features characteristics of nucleosomes remodeled by RSC. A. IS percentiles plotted against nucleosomes that overlap with genomic features, as indicated. **B**. Strongly remodeled nucleosomes plotted against the area around the TSS. **C**. Mean GC content across the different RS percentiles compared to that of the genome average. **D**. Average fraction of nucleosomes covered by poly(dA:dT) tracts across the different RS percentiles compared to that of the genome average. **E**. Histone marks detected by (Pokholok et al., 2005) at genes and overall, across the different RS percentiles.

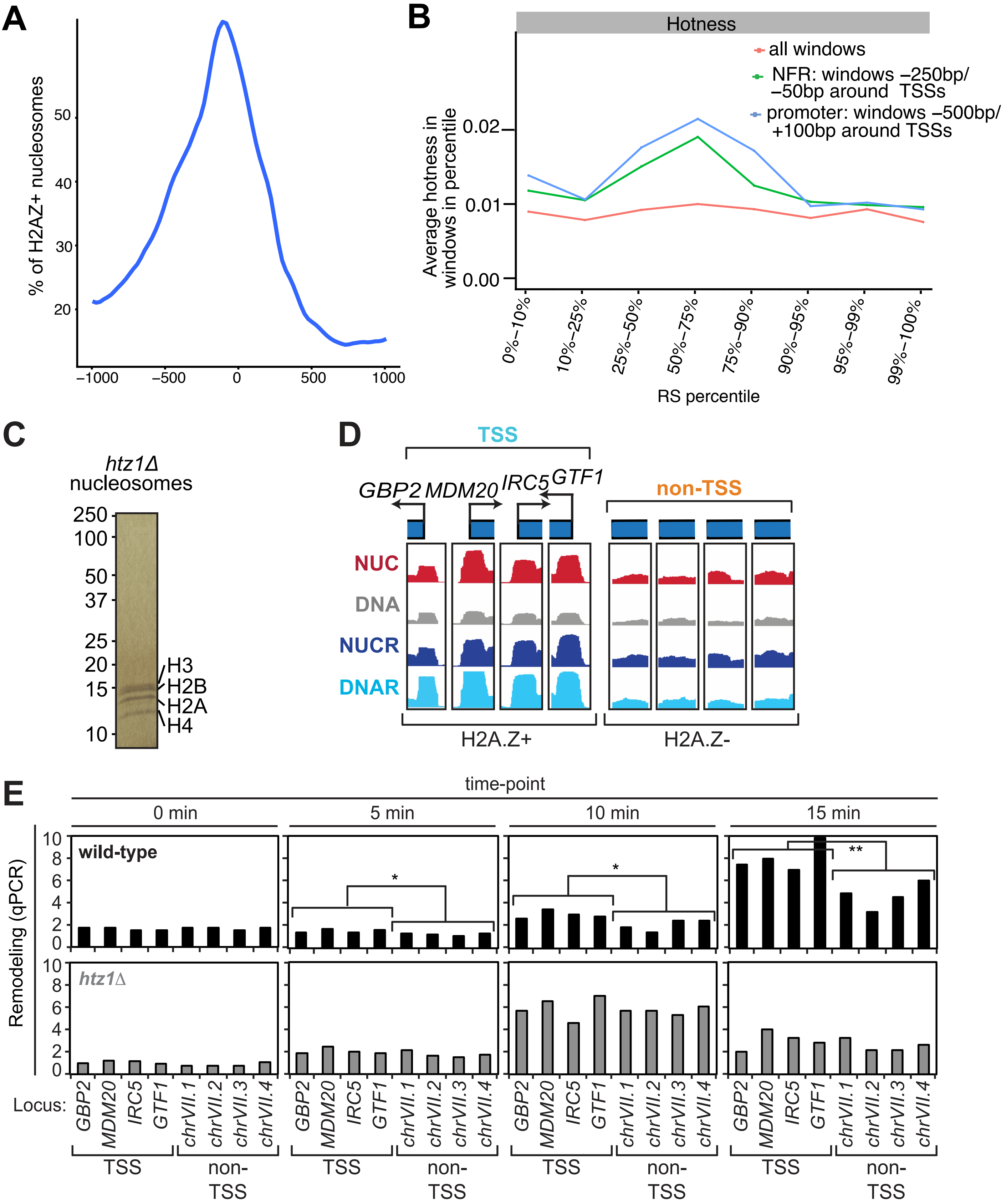

Figure S5. H2A.Z is characteristics of nucleosomes remodeled by RSC. A. H2A.Z-containing nucleosomes and their position around the TSS of genes, using data from (Albert et al. 2007). B. Nucleosome hotness across the different RS percentiles, at NFRs and promoters compared to that of the genome average, using data from (Dion et al. 2007). C. Nucleosomes used for qPCR analysis. D. Nucleosomes used for q-PCR experiments on the effect on RSC remodeling of containing high (H2A.Z+) or low (H2A.Z-) H2A.Z density. E. Data for Figure 5C decomposed into individual nucleosomes. Note that for the experiments in Figure 5C, nucleosomes that are not on the TSS were used as controls, given that (1) the background activity of RSC on all nucleosomes is already high, and (2) many TSS's that we call as lacking H2A.Z might actually carry enough H2A.Z to stimulate catalysis, since nearly all genes have been reported to carry some H2A.Z on their promoters (Raisner et al. 2005; Albert et al. 2007).

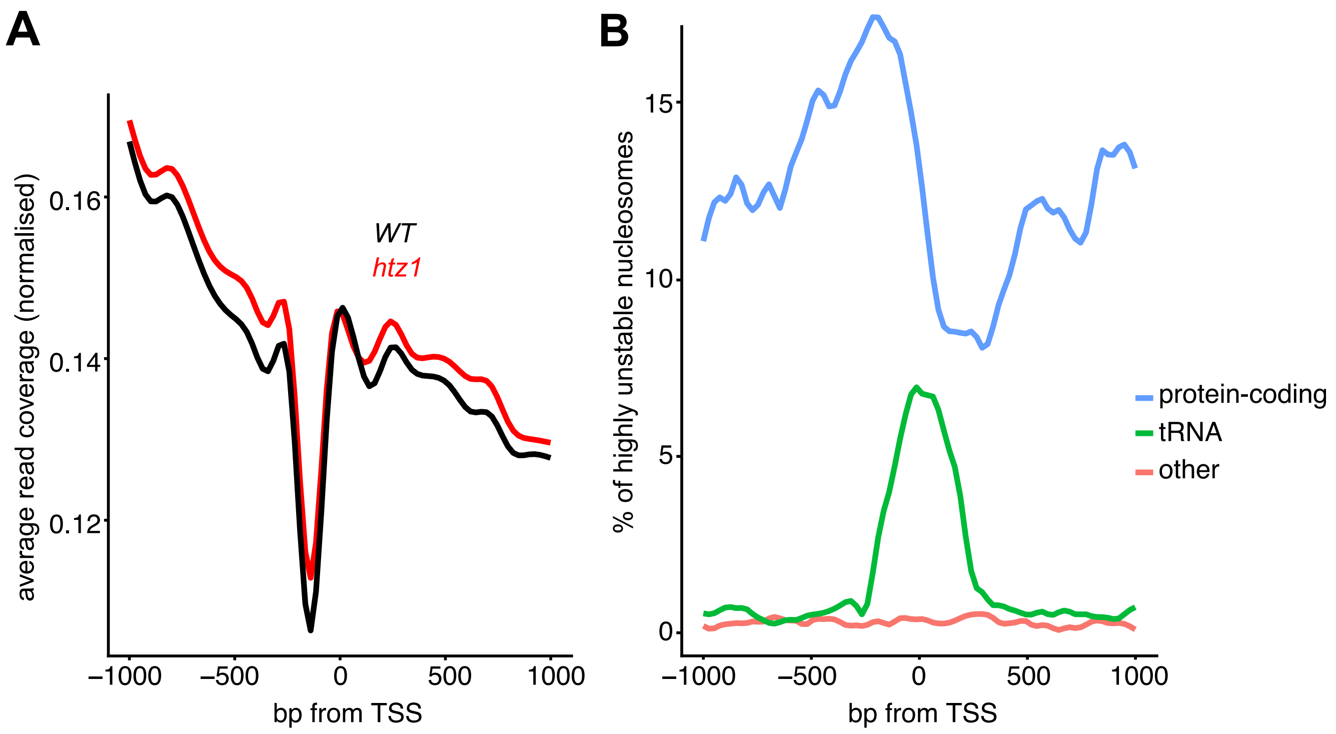

Figure S6. Characteristics of nucleosome libraries from htz1∆ cells. A. Average read coverage comparison between repeat libraries from WT and htz1∆. B. Profile of nucleosome occupancy in the 99th IS percentile, around the TSS of different gene types. Note the overall similarity to wild-type (Figure 3C).
